# “Six-and-Twelve” Prognostic Score in Transarterial Chemoembolization-Treated Hepatocellular Carcinoma Patients

**DOI:** 10.7759/cureus.49575

**Published:** 2023-11-28

**Authors:** Johan S Lopera Valle, Daniel F Puello Correa, Emilio Sanín

**Affiliations:** 1 Interventional Radiology, San Vicente Fundación University Hospital, Medellín, COL; 2 Interventional Radiology, San Ignacio University Hospital, Bogotá, COL; 3 Interventional Radiology, Pablo Tobón Uribe Hospital, Medellín, COL

**Keywords:** pronostic, interventional radiology guided embolization, survival, therapeutic chemoembolization, hepatocellular carcinoma

## Abstract

Objective: This study aimed to evaluate the overall survival of hepatocellular carcinoma patients who qualify for transarterial chemoembolization (TACE) using the "six-and-twelve" prognostic score. The research was conducted on a patient cohort between 2009 and 2019.

Materials and methods: A retrospective cohort study was conducted, involving patients diagnosed with unresectable hepatocarcinoma, Barcelona* *Clinic Liver Cancer (BCLC) staging A or B, and Child-Pugh staging A or B. Exclusion criteria included patients with spontaneous tumor rupture, other neoplasms, decompensated liver cirrhosis, and a lack of reference images. The study assessed the size of the largest nodule and the number of tumors based on imaging studies. Overall survival was defined as the time from initial TACE to death from any cause, with telephonic follow-up conducted. Patients were categorized into three groups based on tumor burden: ≤6, >6-≤12, and >12. Mortality rates at 12, 24, and 36 months were compared using the chi-square test for categorical variables and the ANOVA and Kruskal-Wallis tests for continuous variables, depending on their distribution.

Results: A total of 90 patients were included in the study, with a median age of 69 years (interquartile range (IQR): 62-77). Among the patients, 61.1% had a tumor burden of six or less. The overall survival rate was found to have a median of 28.4 months (IQR: 26.3-30.5), with survival rates at one, two, and three years being 84.7%, 55.2%, and 29.4%, respectively. It was observed that mortality increased in proportion to tumor burden, and this difference was statistically significant.

Conclusion: The use of tumor burden, with cut-off points of six and 12, as a prognostic score proved to be a valuable tool for predicting mortality in the studied cohort.

## Introduction

Hepatocellular carcinoma (HCC) is the sixth most common cancer and the fourth leading cause of cancer-related mortality worldwide, with an overall survival rate of 18% over five years [1].

According to the guidelines of the American Association for the Study of Liver Diseases (AASLD) and the European Association for the Study of the Liver (EASL), transarterial chemoembolization (TACE) is currently the only recommended treatment option for patients with intermediate-stage HCC [2]. This treatment is suitable for patients with preserved liver function and functional status [3,4].

Transarterial chemoembolization is considered for patients in the early stages with tumors that are deemed unresectable due to factors such as size, location, and patient age [5,6]. However, this patient population is heterogeneous, with a median overall survival ranging from 13 to 43 months [7]. Therefore, it is crucial to develop a risk stratification tool [8].

To address this need, Wang et al. [9] developed the "six-and-twelve" prognostic score in 2019. This score, based on tumor burden, serves as an easy-to-use tool for stratifying TACE candidates (Barcelona Clinic Liver Cancer (BCLC) staging A or B) and predicting their survival with favorable accuracy. The prognostic score was derived from a population of Chinese ethnicity where chronic hepatitis B is the predominant cause, which differs from our population where alcoholic and non-alcoholic steatohepatitis are the main causes [10].

The objective of this study was to evaluate the overall survival of HCC patients eligible for TACE based on the "six-and-twelve" prognostic score. The study was conducted on a cohort of patients from Hospital Pablo Tobón Uribe, Medellín, Colombia, spanning from 2009 to 2019.

## Materials and methods

Study population

This retrospective cohort study included patients diagnosed with HCC through imaging or histology, following the guidelines of AASLD or EASL. The patients included were those with unresectable HCC categorized as BCLC staging A and B, based on criteria such as preserved liver function, good functional status, and the absence of vascular invasion or extrahepatic spread. Stage A included patients with a single nodule larger than 2 cm or up to three nodules equal to or smaller than 3 cm, while stage B referred to multinodular HCC. Additionally, patients without prior HCC treatment, with a Child-Pugh score of A5-B7, and with at least one measurable lesion larger than 1 cm were included.

Patients with spontaneous tumor rupture, comorbidity with other malignant neoplasms, decompensated liver cirrhosis (gastrointestinal bleeding, ascites, jaundice, or encephalopathy), a performance status (PS) score greater than 0, previous treatment with systemic or locoregional therapy, and a lack of reference imaging information were excluded.

One investigator determined the size of the largest nodule (tumor size, measured in centimeters) and the number of tumors using multiphase computed tomography (CT) or dynamic contrast-enhanced magnetic resonance imaging (MRI). The tumor burden was calculated by adding the size of the largest tumor (in centimeters) to the number of tumors.

Transarterial chemoembolization

Informed consent was obtained prior to the procedure. The common femoral or right radial artery was punctured using a micropuncture set under an aseptic technique, local anesthesia, and Doppler ultrasound guidance. A short 5-Fr introducer was then advanced through the puncture site. Fluoroscopic guidance was used to access the superior mesenteric artery for indirect portography using a diagnostic catheter and hydrophilic guide. Hepatic arteriography was subsequently performed to document hypervascular lesions. Chemoembolization with doxorubicin (20-50 mg) and 100-300 micron microspheres was carried out by selectively catheterizing branches of the hepatic artery using a 2.8 Fr microcatheter until stasis was achieved.

The TACE sessions were scheduled at intervals of four to six weeks for patients with favorable evolution, as determined by CT or MRI demonstrating tumor viability or intrahepatic recurrence. Factors considered included clinical and laboratory findings (functional status, liver function, etc.), as well as the absence of extrahepatic dissemination or vascular invasion. All procedures were performed by interventional radiologists with a minimum of 10 years of experience.

Statistical analysis

Medical records were reviewed to identify clinical and laboratory variables for inclusion in the study. Overall survival was defined as the time between initial TACE and death from any cause. Telephonic follow-up was conducted to verify survival and determine the date of death, if applicable. Patients lost to follow-up were excluded.

IBM SPSS software version 22 (IBM Corp., Armonk, NY) was used for the analysis, in accordance with the proposed objectives. Descriptive statistics such as absolute and relative frequencies were used to describe qualitative variables, while mean and standard deviation or median and interquartile ranges were used for quantitative variables, depending on their distribution in the study population.

According to the prognostic score "six-and-twelve" developed by Wang et al. in 2019 [9], patients were divided into three groups based on tumor burden (≤6, >6 -≤12, and >12). Mortality at 12, 24, and 36 months was then compared between these groups. Hazard ratios (HR) and their respective 95% confidence intervals were calculated using the Cox regression model.

Ethics statement

The research obtained approval from the Ethics Committee of Pablo Tobón Uribe Hospital, Medellín, Colombia, and was conducted in adherence to ethical principles for research, following the Declaration of Helsinki and Resolution 008430 of 1993 from the Ministry of Health of Colombia.

## Results

**Figure 1:**
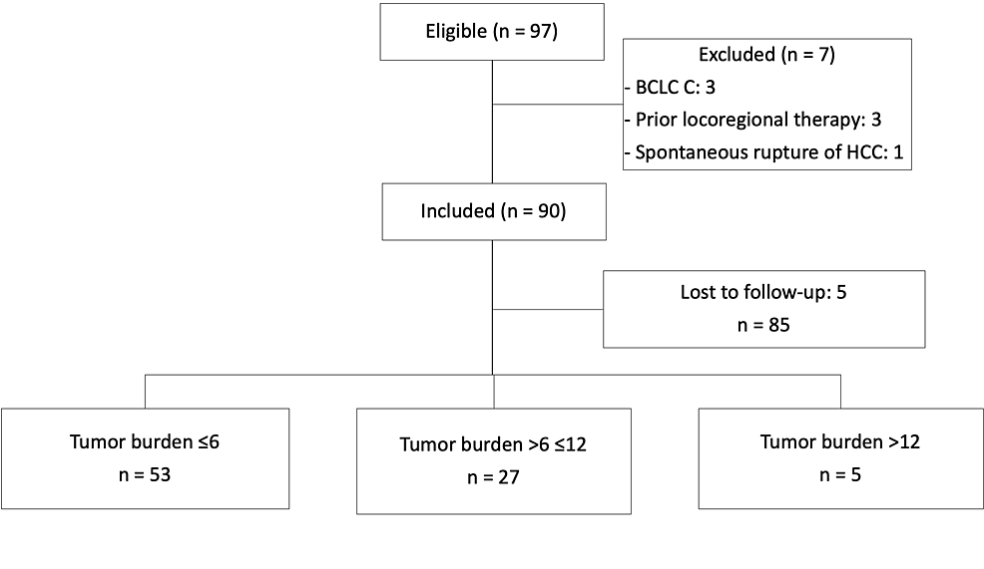
Flow chart of the patient selection BCLC: Barcelona Clinic Liver Cancer staging system; HCC: hepatocellular carcinoma

Out of 97 eligible patients, a total of 90 were included. The median age was 69 years (interquartile range (IQR): 62-77), and 60.8% of the patients were men (n=59) (Figure 1).

The most common cause of cirrhosis was alcoholic liver disease, followed by cryptogenic cirrhosis and non-alcoholic steatohepatitis, accounting for 31.1%, 26.6%, and 23.3%, respectively. The median size of the main tumor was 4.2 cm (IQR: 3-5.7), and 64.4% of patients had a size greater than 3 cm but less than or equal to 7 cm. Just over half of the patients (56.6%) had a single tumor, with an average of 1.5 tumors (SD: 0.8-2.2); 61.1% of patients had a tumor burden of six or less, while the mean tumor burden for the entire group was 6.4 (SD: 5.7-8.1); 70% of patients had an alpha-fetoprotein (AFP) level lower than 400 ng/ml; 55.6% were classified with Child-Pugh score B; and 73.4% were classified as BCLC stage B (Table 1).

**Table 1:** Clinical characteristics of transarterial chemoembolization-treated hepatocellular carcinoma patients BCLC: Barcelona Clinic Liver Cancer staging system

Clinical characteristics, n= 90	n (%)
Etiology	
Alcoholic liver disease	28 (31.1)
Cryptogenic	24 (26.6)
Non-alcoholic steatohepatitis	21 (23.3)
Hepatitis virus	13 (14.4)
Non-cirrhotic liver	5 (5.5)
Largest tumor diameter (cm)	
≤3	17 (18.8)
>3-≤7	58 (64.4)
>7-≤10	8 (8.8)
>10	7 (7.7)
Number of tumors	
1	51 (56.6)
2	29 (32.2)
3	9 (10)
4	1 (0.9)
Tumor burden	
≤6	55 (61,1)
>6-≤12	28 (31.1)
>12	7 (7.7)
Alpha-fetoprotein, ng/ml	
<400	63 (70)
≥400	27 (30)
Child-Pugh score	
A	49 (54.4)
B	41 (55.6)
BCLC staging	
A	33 (36.6)
B	57 (73.4)
Transplant during follow-up	14 (16.4)

The median number of TACE sessions was two (IQR: 1-2). The median follow-up period was 20.5 months (IQR: 12-38.2), with a range of four to 124 months. Five patients were lost to follow-up and were excluded from the survival analysis (N=85). Table 2 compares the follow-up groups based on tumor burden and other clinical variables of interest, but no statistically significant differences were observed.

**Table 2:** Comparison between follow-up groups BCLC: Barcelona Clinic Liver Cancer staging system; TACE: transarterial chemoembolization

Clinical characteristics, n= 90	Tumor burden			p-value
	≤6	>6 ≤12	>12	
Age, median	67.2	70.2	69.8	0.23
Male,%	57.2	59.3	60.5	0.26
Alpha-fetoprotein ≥400 ng/ml, %	28.2	30.2	31	0.31
Child-Pugh score B, %	54	55.1	55.9	0.18
BCLC B, %	71	72.9	74.3	0.19
TACE sessions, median	1.5	2	2.5	0.09

**Figure 2:**
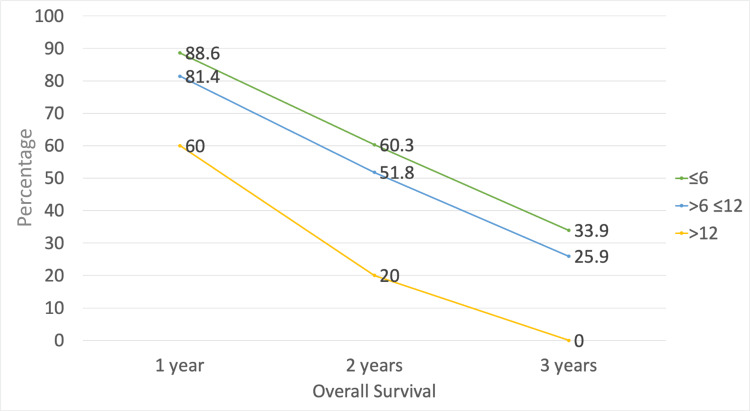
Percentage of overall survival according to tumor burden Different lines ≤6, >6 ≤12, and >12 represent tumor burden

The present cohort (n=85) had an overall survival median of 28.4 (IQR: 26.3-30.5) months. The one-, two-, and three-year survival rates were 84.7%, 55.2%, and 29.4%, respectively. Tumor burden directly influenced overall survival, with one-year survival rates of 88.6% versus 60% in burdens ≤6 and >12, respectively (HR=1.64; 95% CI: 1.23-2.05; p=0.021). Similarly, two-year survival rates were 60.3% versus 20% in burdens ≤6 and >12, respectively (HR=1.89; 95% CI: 1.51-2.27; p=0.015), and three-year survival rates were 33.9% versus 0% in burdens ≤6 and >12, respectively (HR=3.11; 95% CI: 2.84-3.38; p=0.0001) (Table 3 and Figure 2).

**Table 3:** Overall survival according to tumor burden IQR: interquartile range

Tumor burden	n	Overall survival in months, median (IQR)	1 year, n (%)	2 years, n (%)	3 years, n (%)
≤6	53	31.6 (27.3-35.9)	47 (88.6)	32 (60.3)	18 (33.9)
>6-≤12	27	21.8 (18.2-25.4)	22 (81.4)	14 (51.8)	7 (25.9)
>12	5	14.3 (9.1-19.5)	3 (60)	1 (20)	0
Total	85	28.4 (26.3-30.5)	72 (84.7)	47 (55.2)	25 (29.4)

## Discussion

In 2019, Wang et al. [9] developed the "six-and-twelve" score based on a multicenter cohort of 1,604 patients undergoing TACE (BCLC staging A or B). This score predicted individual prognosis by calculating tumor burden and showed favorable performance. They identified three strata (≤6, >6-≤12, and >12) based on tumor burden alone for survival prediction.

Unlike Wang et al.'s study [9], our study included various etiologies of HCC rather than being predominantly focused on hepatitis B virus infection (85.2%). This reflects the diverse patient population with cirrhosis and HCC encountered in daily clinical practice in the West.

The overall survival of the present cohort had a median of 28.4 months, similar to the 32.9 months reported by Wang et al. [9]. Reporting slightly lower values, Lencioni et al. [11] and Kaewdech et al. [12] described median survivals of 19.8 and 20.3 months, respectively. On the other hand, according to the 2018 EASL guidelines, the median described for this group of patients was 40 months in well-selected candidates [8].

Thus, as previously stated, the overall survival of our cohort was directly proportional to the tumor burden, being higher in patients with a burden of six or less and significantly lower in those with a burden greater than 12. Median survivals of 31.6, 21.8, and 14.3 months were described according to tumor burden ≤ 6, >6-≤12, and >12, respectively. When compared to the available literature, Kaewdech et al. [12] validated the "six-and-twelve" score in 716 patients undergoing TACE. They described similar median survivals of 35.1, 16, and 7.6 months for the ≤6, >6-≤12, and >12 groups, respectively. Wang et al. [9], on the other hand, described higher survivals for the ≤6 and >6-≤12 groups (49.1 and 32.0 months, respectively) and similar survival in the >12 tumor burden group (15.8 months).

The reasons that could explain lower survivals in our cohort compared to those reported by Wang et al. [9] in China could be related to the different etiologies of cirrhosis, as well as the lower proportions of BCLC-A HCC and Child-Pugh A scores in our population. It is known that patients with HCC associated with the hepatitis B virus have better survival than those with other etiologies [13]. In addition, BCLC-A tumor stage and preserved liver function (Child-Pugh A score) are also important prognostic predictors.

The median survival of the three strata identified by the "six-and-twelve" score varies within a range of 13-43 months according to various studies, according to tumor burden [11-13], which demonstrates that this population undergoing TACE has a different prognosis even when liver function and functional status are preserved. This supports the need for and importance of risk stratification for these patients in clinical practice, reducing heterogeneity in turn.

The "six-and-twelve" score was specifically developed for ideal TACE candidates, and cutoff values based on evidence were adopted based on a large multicenter cohort, constituting an accurate score for risk stratification. Previously described scores, such as the "four-and-seven" score and the hepatoma arterial-embolization prognostic (HAP) score, are aimed at all patients treated with TACE, and the inclusion of cases beyond current recommendations may influence patient characteristics and heterogeneity profiles. The application of the "six-and-twelve" score, on the other hand, is aimed at patients with unresectable HCC, preserved liver function, and acceptable functional status, without exclusion criteria based on tumor size or number. In this population, tumor burden is a fundamental prognostic factor, regardless of systemic or locoregional treatment [14-16].

The "six-and-twelve" score is easily applicable and allows for a rapid evaluation based on imaging studies, regardless of AFP level, liver function, or etiology.

The "six and twelve" model provides an estimated probability of survival and a median survival as a reference for patients with unresectable HCC, who are ideal candidates for TACE in our setting. This information helps in making decisions about other treatment options. For instance, the current scoring system indicates that patients with combined tumor size and number exceeding 12 have a median survival of 14.3 months, which is shorter than that of intermediate-stage patients receiving sorafenib due to being unfit or unresponsive to locoregional treatment [17,18]. This suggests that although it is assumed that this population would benefit from TACE, the reported survival for patients with a higher tumor burden may be modest, and the decision to perform exclusive TACE should be carefully considered.

In the validation carried out by Kaewdech et al. [12], the validity of the "six-and-twelve" score in predicting the survival rate of unresectable HCC treated with TACE was confirmed. However, the area under the receiver operating characteristic (ROC) curve (AUROC) of the "six-and-twelve" score in that validation cohort was slightly lower compared to the original Chinese cohort. The authors also conclude that although the "six-and-twelve" score is currently the most predictive, it may not be ideal for predicting survival in HCC patients treated with TACE, as the AUROC falls within an acceptable but not excellent range. They suggest that additional studies should be conducted to develop new scores with higher predictive performance.

Our study has notable strengths. Firstly, it reflects the daily clinical practice where the etiology of HCC is heterogeneous, unlike the population in the original "six-and-twelve" score. Secondly, it is the first study conducted with the "six-and-twelve" score in a Latin population.

There are some limitations to mention. First, the risk of selection bias is inevitable in this type of observational study. However, we attempted to minimize it by consecutively including all patients. Second, this study does not validate the "six-and-twelve" score, which is necessary to conclusively determine its utility in our population. On the other hand, although not considered in the "six-and-twelve" score, liver function parameters as a prognostic factor may be crucial in patients with other etiologies of cirrhosis who are more prone to liver function deterioration after TACE [3]. Lastly, treatment response has not been considered an important parameter in determining prognosis. However, in daily clinical practice, the performance of imaging studies can delay the timing of stratification and the timely conduct of TACE sessions.

## Conclusions

Transarterial chemoembolization is considered for patients in the early stages who are deemed unresectable due to factors such as tumor size, location, and age. However, the heterogeneity of these patients necessitates the implementation of prognostic tools for precise therapeutic approaches. The "six-and-twelve" score is easily applicable and enables an evaluation based on imaging studies, irrespective of other clinical values like liver function or etiology, to ensure accurate patient selection prior to the initial TACE. The "six-and-twelve" score can be useful in evaluating clinically significant outcomes in daily practice and is also an important tool in designing clinical trials with comparable criteria and stratified risk.
